# Tumor Endothelial Cell—A Biological Tool for Translational Cancer Research

**DOI:** 10.3390/ijms21093238

**Published:** 2020-05-03

**Authors:** Dorcas Akuba-Muhyia Annan, Hiroshi Kikuchi, Nako Maishi, Yasuhiro Hida, Kyoko Hida

**Affiliations:** 1 Department of Vascular Biology and Molecular Pathology, Hokkaido University Graduate School of Dental Medicine, Sapporo 060-8586, Japan; annandorcasam@gmail.com (D.A.-M.A.); mnako@den.hokudai.ac.jp (N.M.); 2 Department of Renal and Genitourinary Surgery, Hokkaido University Graduate School of Medicine, Sapporo 060-8638, Japan; hiroshikikuchi16@yahoo.co.jp; 3 Department of Cardiovascular Thoracic Surgery, Hokkaido University Faculty of Medicine, Sapporo 060-8638, Japan; yhida@med.hokudai.ac.jp

**Keywords:** tumor endothelial cell, translational research, antiangiogenesis drugs

## Abstract

Going from *bench to bedside* is a simplified description of translational research, with the ultimate goal being to improve the health status of mankind. Tumor endothelial cells (TECs) perform angiogenesis to support the growth, establishment, and dissemination of tumors to distant organs. TECs have various features that distinguish them from normal endothelial cells, which include alterations in gene expression patterns, higher angiogenic and metabolic activities, and drug resistance tendencies. The special characteristics of TECs enhance the vulnerability of tumor blood vessels toward antiangiogenic therapeutic strategies. Therefore, apart from being a viable therapeutic target, TECs would act as a better mediator between *the bench* (i.e., angiogenesis research) and *the bedside* (i.e., clinical application of drugs discovered through research). Exploitation of TEC characteristics could reveal unidentified strategies of enhancing and monitoring antiangiogenic therapy in the treatment of cancer, which are discussed in this review.

## 1. Introduction

Basic research provides a wealth of information to explain scientific and clinical observations; however, at this level, nothing can be done clinically to improve the health of patients if the research findings are not applied appropriately. Translational research is one important strategy to bridge this gap. According to the Evaluation Committee of the Association for Clinical Research Training (ACRT), “translational research fosters the multidirectional integration of basic research, patient-oriented research, and population-based research, with the long-term aim of improving the health of the public” [1]. There are three levels of translational research (i.e., T1, T2, and T3) which have a cyclical relationship because research is continuous. This review addresses the T1 level (“which advances the movement between basic research and patient-oriented research that leads to new or improved scientific understanding or standards of care” [1]) with regard to cancer therapy via tumor angiogenesis research. Angiogenesis research is well defined in the field of basic science, and the development of antiangiogenic agents has carried the importance of this field into the clinical setting to manage and/or inhibit all types of pathological angiogenesis, including tumor angiogenesis. The majority of growing tumors thrive on angiogenesis and other mechanisms to establish tumor vasculature. Through the process of angiogenesis, the growing tumor is provided with blood vessels, without which the tumor will remain as a small mass of cells less than 2 mm in diameter [2]. Therefore, tumor angiogenesis has been a pivotal target for cancer therapy. Various antiangiogenesis drugs/angiogenesis inhibitors and targetable molecules are being identified every so often. However, the complexity of using antiangiogenesis drugs poses a challenge, that is, the positive benefits of the antiangiogenesis drugs make patients hopeful, whereas the detrimental side effects leave clinicians conflicted. Consequently, antiangiogenic therapy has become a two-edged treatment strategy, which must be fine-tuned to maximize the therapeutic benefits and gradually diminish the negative side effects. Tumor endothelial cells (TECs), being distinct from normal endothelial cells (NECs), possess characteristics and features that are useful in translational research for the improvement of cancer treatment. This review discusses how TECs can serve as a better tool in translational research.

## 2. Tumor Vasculature

Tumors become vascularized through more than one mechanism of angiogenesis. It may take the form of sprouting angiogenesis [3] from preexisting vessels or the splitting of preexisting vessels into two daughter vessels by a process known as intussusception [4]. Neovascularization processes such as vasculogenesis mediated by endothelial progenitor cells (EPCs) recruited from the bone marrow can lead to the development of tumor blood vessels [5]. In addition, through the process of vasculogenic mimicry, highly invasive and metastatic melanoma cells mimic the endothelium-forming ability of endothelial cells (ECs) and create loops or networks resembling the vasculature, which are devoid of ECs but contain blood cells [6]. These channels facilitate tumor blood supply independent of angiogenesis. Breast, colon, lung, pancreatic, ovarian, glioblastoma multiforme, and hepatocellular carcinomas are among the cancer types that present with vasculogenic mimicry [7]. 

**Figure 1:**
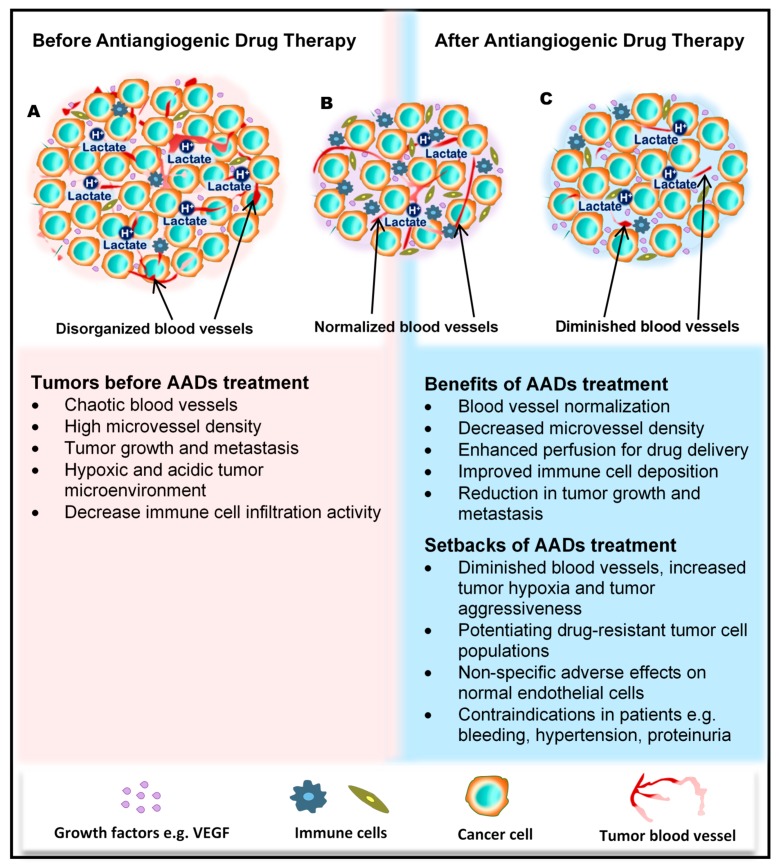
Benefits and side effects of antiangiogenic drugs. AADs, antiangiogenesis drugs. The dependency of tumors on their resident blood vessels to grow and metastasize has led to the targeting of tumor blood vessels to starve the tumor cells and close the metastasis portals. (**A**) Before the administration of AADs, the tumor histology is characterized by a high density of microvessels, with an undefined order of organization. The microenvironment is generally acidic, with high lactate levels, and immunologically suppressed. (**B**) However, after AAD therapy, tumor blood vessels become normalized, microvessel number reduces, tumor growth recedes, and immune cells infiltrate the tumors more through the normalized vasculature. (**C**) In addition to these benefits, AAD use causes some undesirable effects, including tumor hypoxia (from prolonged use of AADs) and destruction of normal vessels leading to bleeding. Patients may also experience hypertension and proteinuria, among others.

The tumor blood vessels carry nutrients to the tumor to stimulate rapid growth of the tumor, enrich the stroma with immune cells, and also aid tumor metastasis. In the wake of their development, tumors cause significant transformations in all cells and tissues in their surroundings. The growing tumor begins to exert physical pressure on the vessels, thus causing portions of the vessels to flatten and lose their lumen. Hierarchal vessel structure and blood flow are distorted (Figure 1A). Moreover, tumor-derived growth factors such as vascular endothelial growth factor (VEGF) stimulate rapid angiogenesis without sufficient control from angiogenesis inhibitors, which leads to the formation of tortuous vessels with loose EC junctions [8], little or no perivascular cell coverage [9], and an overall leaky nature, further contributing to the high interstitial fluid pressure observed in tumors [10,11].

## 3. Angiogenesis and Its Inhibition in Tumors

### 3.1. Angiogenesis

Sprouting angiogenesis is the physiological process that was described as the formation of new blood vessels by capillary sprouting from preexisting vessels. Most blood vessels remain quiescent in the adult body, and angiogenesis occurs only in female reproductive organs and in the placenta during pregnancy. However, ECs preserve the function of rapid division in response to a physiological stimulus such as hypoxia or inflammation [12]. Angiogenic factors such as VEGF, basic fibroblast growth factor (bFGF), angiopoietin, and platelet-derived growth factor (PDGF) drive angiogenesis, and it is also performed as a normal process in growth and developmental processes, such as wound healing [13,14].

### 3.2. Factors That Stimulate and Regulate Tumor Angiogenesis

The tumor uses existing angiogenic mechanisms to induce capillary growth. Various growth factors, including VEGF, bFGF, PDGF, and angiopoietin, can induce tumor angiogenesis [15]. These factors are secreted from tumor cells and stromal cells. For example, tumors activate tumor-associated macrophages (TAMs) or neutrophils to produce angiogenic factors such as VEGF and matrix metalloproteinases (MMPs) [16,17]. Furthermore, other immune cell types indirectly influence the process of angiogenesis through the secretion of VEGF-A, bFGF, MMP9, interferon gamma (IFNγ), and interleukin-17 (IL-17) [18,19,20]. These angiogenesis stimuli may also be triggered by metabolic stress such as hypoxia, low pH or hypoglycemia, mechanical stress and genetic mutations, and p53 regulation [21,22,23,24].

### 3.3. Concept of Antiangiogenic Therapy and Development of Angiogenesis Drugs and Their Molecular Targets

For a long period of time, cytotoxic drugs were conventionally used for anticancer treatment. Later on, antiangiogenic therapy was proposed as a new concept for anticancer treatment. Dr. Judah Folkman in the early 1970s proposed that cancer could be treated by blocking the supply of oxygen and nutrients through the inhibition of tumor angiogenesis [25]. Moreover, antiangiogenic therapy has the potential to normalize blood vessel structures and improve systemic delivery of oxygen or perfusion of cytotoxic drugs into tumor tissues (Figure 1B) [26,27].

Bevacizumab, a monoclonal antibody targeting VEGF, was first approved as an antiangiogenic therapy in 2004 for the treatment of colon cancer in combination with chemotherapy [28,29]. Since then, various antiangiogenesis drugs, either as monotherapy or in combination with other cytotoxic drugs, have been developed, used in clinical trials, and approved for the treatment of cancer. These antiangiogenesis drugs include tyrosine kinase inhibitors (TKIs) such as sorafenib, sunitinib, axitinib, and pazopanib target receptors for VEGF and PDGF to inhibit the VEGF pathway [30]. 

Besides VEGF and its receptors, several other growth factors and receptors are involved in pathways that regulate tumor growth and angiogenesis in a complementary and coordinated manner. New multikinase inhibitors that can simultaneously target more than one of these pathways have been developed and approved for anticancer treatment. For example, regorafenib was found to inhibit a distinct set of receptor kinases, including the vascular endothelial growth factor receptors (VEGFR1–3), TIE2, fibroblast growth factor receptor 1 (FGFR1), and platelet-derived growth factor receptor beta (PDGFR-b), and has been approved for treating hepatocellular carcinoma, colorectal cancer, and gastrointestinal stromal tumors [31,32,33,34]. Cabozantinib was found to inhibit VEGFR2, c-Met, and AXL receptor tyrosine kinases and has been approved for treating metastatic renal cell carcinoma [35,36]. Several other multikinase inhibitors have also been developed and used in clinical trials or clinical settings [37]. The mammalian target of the rapamycin (mTOR) pathway is also involved in angiogenesis, and mTOR inhibitors such as everolimus and temsirolimus have been approved for clinical use [38].

### 3.4. Positive Achievements and Clinical Outcomes of Antiangiogenic Therapy

Antiangiogenic therapy enhances T-cell priming and activation by promoting dendritic cell maturation and increasing T-cell infiltration into the tumor tissue via tumor vessel normalization [39,40,41]. Antiangiogenic therapy also converts an immune-permissive tumor microenvironment by decreasing regulatory T-cell and myeloid-derived suppressor cells [41,42]. Tumor vessel normalization is crucial for improving the immune environment, tumor immunity is the key factor for anticancer treatment, and improving the immune environment is necessary to increase treatment efficacy (Figure 1B). Today, immune checkpoint inhibitors have been approved for treating various cancers [43,44,45]; furthermore, recent therapeutic strategies target both tumor angiogenesis and tumor immunity for achieving a greater therapeutic effect. As a combination therapy, bevacizumab and IFNα have been approved for treating metastatic renal cell carcinoma [46]. Clinical trials of combined TKIs and immune checkpoint inhibitors are actively being conducted, and favorable outcomes have been observed in metastatic renal cell carcinoma and non-small cell lung cancer [47,48,49,50].

### 3.5. Negative Side Effects/Adverse Responses from Patients

Regardless of the enormous benefits, antiangiogenic therapy has several problems. Complications such as hypertension, hand–foot syndrome, proteinuria, and thyroid dysfunction could occur as adverse events because of the effect of antiangiogenesis drugs on normal blood vessels [51]. Drug resistance to antiangiogenic therapy may also occur, and drug switching is generally required. Various mechanisms are described for explaining the resistance to antiangiogenic therapy, and various cellular and noncellular factors in the tumor microenvironment such as TECs, immune cells, cancer-associated fibroblasts, or extracellular matrix components are involved in these resistance mechanisms [52,53]. Long-term antiangiogenic therapy leads to tumor hypoxia and induces tumor aggressive behavior [54] (Figure 1C). It has been reported that hypoxia promotes the accumulation of TAMs [55] and induces tumor angiogenesis through the mobilization of bone marrow-derived endothelial precursor cells [56]. Hypoxia also induces chromosomal abnormalities in TECs via reactive oxygen species [57] and the selection of more invasive metastatic populations of tumor cells that are resistant to antiangiogenic therapy [58] (Figure 1C). Other modes of tumor vascularization such as vascular mimicry and vessel co-option have been suggested as another mechanism underlying the resistance to antiangiogenic therapy. Vascular mimicry is a tumor blood supply system without the participation of ECs. Vascular-like constructions are generated through the differentiation of tumor cells into endothelial-like cells, independent of conventional angiogenic factors [6]. An increase in vascular mimicry has been observed after antiangiogenic therapy [59]. Vessel co-option occurs in metastatic tumors. Tumor cells co-opt and grow around existing blood vessels [60]. Vessel co-option could explain the cause of resistance to antiangiogenic therapy in various cancers [61,62,63].

## 4. Tissue and Cellular Sources of TECs

### 4.1. Blood Vessels

The key players in the formation of blood vessels and the likely target of antiangiogenic therapy, ECs, through the process of sprouting angiogenesis, migrate and proliferate to form vessels by relying on cues from the growing tumor. Like the vessels recruited into the tumor, the ECs are similarly imparted by the growing tumor themselves as well as the microenvironmental factors, leading to the development of unique characteristics in these recruited ECs different from those of NECs. These endothelial cells, often designated as TECs or tumor-associated ECs, have their primary origin as the blood vessels within the tumor mass. TECs have been isolated from various tumors, and analyses have shown that they indeed have an endothelial lineage [64]; however, some studies have demonstrated that other cellular sources of the tumor endothelium exist. 

### 4.2. Cancer Stem Cells and EPCs

Cancer stem cells (CSCs) and EPCs are involved in this nonconventional tumor vasculature formation through vasculogenesis. ECs in glioblastoma were found to have similar genetic alterations as those in the tumor cells; moreover, glioblastoma stem cells positive for the stem cell marker CD133 were capable of generating cells that phenotypically and functionally resembled ECs [65,66]. Contrary to the above suggestions that TECs could arise from cancer stem cells, some recent reports have demonstrated that it is rather rare to find ECs with neoplastic origins within the glioblastoma vasculature. Kulla et al. reported that glomeruloid vessels microdissected from glioblastoma tissue specimens lacked mutations in the tumor protein p53 (TP53) gene as compared to the surrounding glioblastoma cells with a mutation in 3 exons of this gene [67]. Additionally, epidermal growth factor receptor (EGFR) gene amplification could not be identified in CD34+ endothelial cells within the vascular linings of glioblastoma tissue, further supporting the unlikely contribution of glioblastoma cells to tumor vessel formation [68]. However, glioma stem cells may contribute to tumor angiogenesis by differentiating into perivascular cells, like pericytes, not endothelial cells, which are necessary for blood vessel maturation [69] 

EPCs originate as progenitor cells from the bone marrow possessing the ability to develop into matured ECs. As EPCs travel from the bone marrow into the peripheral blood to the tumors, their surface molecules change from CD133+/CD34+/VEGFR2+ cells to cells with a decreased expression of CD133, while maintaining the expression of VEGFR2 and the hematopoietic progenitor cell antigen CD34 as they mature in circulation. The matured ECs in the vessels finally lose both CD133 and CD34 but display high VEGFR2 expression and are positive for vascular endothelial cadherin, von Willebrand factor, and platelet endothelial cell adhesion molecule 1 (PECAM1), also known as CD31 [70]. EPCs are known to both support vessel formation and integrate directly into the endothelium. Early-forming EPCs secrete angiogenic growth factors and cytokines (e.g., VEGF, stromal cell-derived factor-1 (SDF-1), insulin-like growth factor 1 (IGF-1), and IL-8 [71]) to facilitate neovascularization, whereas late-forming EPCs were found to be better at forming capillary tubes and could differentiate easily into ECs [72]. Both tumor-derived growth factors and EPC-secreted molecules play a role in recruiting EPCs into peripheral circulation and further into the tumor tissue to initiate tumor vasculogenesis. The abundance of EPCs in various types of cancers, including breast cancer [73], non-small cell lung cancer [74], hepatocellular carcinoma [75], colorectal cancer, leukemia, lymphoma, myeloma [76], and glioma [65,66], indicates their relevance in the tumor growth [77]. The role of EPCs in enhancing microvessel formation within some of these tumors has been demonstrated. Therefore, EPCs are an established source of TECs.

## 5. TEC Characteristics

### 5.1. Cytogenetic Abnormality

**Figure 2:**
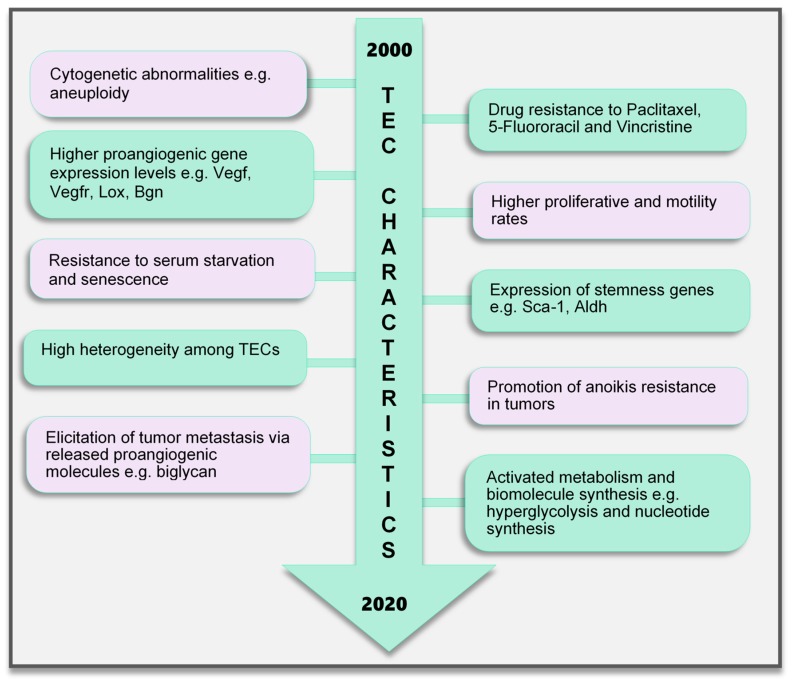
TEC characteristics identified to date. Various characteristics of TECs have been observed, which make them unique when compared with endothelial cells in normal blood vessels. These range from the genetic composition and expression of genes, abnormal karyotype, higher biological activities (proliferation and motility), and TEC influence on tumor cells to modulate cancer cell metastasis and survival to modifications in their metabolic signature. TECs are not normal and their abnormality creates a targetable avenue to influence the growth of tumors and improve therapeutics in cancer treatment. TEC, tumor endothelial cells

Contrary to earlier assumptions about TECs, it has been shown for over a decade that TECs are undoubtedly different from NECs. TECs have characteristics that are considered to be abnormal, ranging from their morphology to genetics and function (Figure 2). TECs obtained from human melanoma and liposarcoma tumor xenografts structurally possessed bigger nuclei with different size variations than those in NECs [78]. These nuclei were made up of chromosomes with various structural aberrations, translocations, chromosomal aneuploidy, missing chromosomes, and the presence of additional chromosomes such as double minutes and some of unknown origin [78]. Microvascular ECs in B-cell lymphoma were also found to have lympho-specific chromosomal translocations [79]. More recently, nonhematopoietic aneuploid CD31^+^ circulating TECs were detected in the peripheral blood of patients with breast cancer, demonstrating that circulating TECs, and not only tumor-bound TECs, possess chromosomal changes [80]. Due to these changes, TECs obtained from human [81] and murine tumors [78] are genetically unstable compared with NECs. We have reported that the TECs isolated from xenograft human epithelial tumors, which were CD133(+), were susceptible to a higher frequency of aneuploidy than the CD133(-) TECs. This suggests that progenitor cells do not only contribute to TEC generation but may also be involved in inducing genetic instability in these cells [81]. Other causes of TEC aneuploidy include hypoxia-induced reactive oxygen species and VEGF signaling [57]. It has been reported that VEGF signaling also regulates the centrosome duplication cycle in ECs and induces centrosome overduplication [82], which could further lead to aneuploidy [83]. Stromal cells like fibroblasts and ECs receive tumor genetic material (DNA/chromosomes) transferred via apoptotic bodies from tumor cells [84,85]. Such horizontal gene transfer can lead to cytogenetic alterations in ECs. Furthermore, genetic instabilities could occur within the cells through the propagation of the DNA. Replication of the transferred DNA may occur in the recipient cells provided they have undergone certain changes including p21 and p53 inactivation [84,85,86]. 

### 5.2. Genotypic Changes

In addition, genes regulating angiogenesis, cell proliferation and motility, stemness, and drug resistance, among others, are altered in TECs. ECs require the autocrine function of VEGF to sustain vascular integrity and viability [87]. VEGF acts through its receptors, the VEGFRs. Among them, VEGFR1 and VEGFR2 have been shown to be highly expressed in TECs compared to those in NECs. This enhances the response of TECs to VEGF more than NECs [88], which makes TECs proangiogenic and may also support their ability to survive in serum-free media unlike their normal counterparts [89]. Furthermore, TECs proliferate and migrate faster than NECs [88]. A study comparing ECs isolated from colorectal cancer and normal colorectal mucosa demonstrated the unique expression of 46 genes in the tumor endothelium (i.e., TECs) as opposed to the normal endothelium [90]. The top 25 genes included MMP2 and MMP11, as well as variations of collagen types I, III, and VI, among others. The authors of that study described these genes as tumor endothelial markers (TEMs) by confirming through the analysis of TEM7 expression in the lung, pancreas, breast, and brain and suggested that the TEMs may be expressed in other cancers as well [90]. These findings established a promising future for the use of the TEMs in further research to identify novel antiangiogenesis strategies. However, later publications suggested that not all the TEMs are unique to TECs. TEM1, TEM 5, TEM7, and TEM 8 were found to be also expressed in normal cells, tissues, and organs [91,92,93,94]. The secreted and membrane forms of TEM7 were observed in various osteogenic sarcoma cell lines [95]. TEC heterogeneity is a major factor contributing to the genotypic differences observed between NECs and TECs. TECs obtained from low-metastatic and high-metastatic tumors have different tendencies toward cell proliferation, motility, and drug resistance, indicating that there may be differences in the underlying genotype of these cells. We had demonstrated that genes required for angiogenesis-related molecules and matrix-degrading enzymes were upregulated in the TECs obtained from highly metastatic tumors [96]. Moreover, the heterogeneity arising from the different cellular origin (stems cells, cancers, and EPCs) of TECs may account for the different genotype of TECs compared with NECs [97]. TECs isolated from different tumor types show variations in their gene expression profiles, the upregulated gene, or the gene set classification. ADAM23, FAP, GPNMB, and PRSS3 genes were high in ovarian cancer ECs [98], while G-protein-coupled receptor RDC1 was the distinctively induced TEM in TECs of the brain and the peripheral vasculature [99]. Furthermore, fewer similarities exist between different TEC gene profiles. A collation of 73 TEC marker genes from five studies showed that at most, only two cancer types shared one of these five genes (EGFL6, HEYL, MMP9, SPARC, PLXDC1), and the rest were expressed uniquely in only one cancer [100]. Aird reviewed that TEC gene expression heterogeneity is influenced by the tumor type, extracellular environment, and epigenetic regulation [100]; however, the current vast database needs to be sifted through by further research to obtain significant translational benefits.

### 5.3. Drug Resistance and Anoikis Resistance

A drug resistance phenotype has been reported in various TECs. The TECs isolated from A375SM (super-metastatic human melanoma cells) xenografts are resistant to the anticancer drug paclitaxel [101], and CD105+ TECs isolated from hepatocellular carcinoma were found to be resistant to doxorubicin and 5-fluorouracil [102]. TEC resistance to paclitaxel was attributed to the upregulated expression of multidrug resistance protein 1 (MDR1) mRNA through VEGF signaling [101]. The ATP-binding cassette (ABC) transporter superfamily, which includes ABCB1 (i.e., MDR1/P-glycoprotein, P-gp), is expressed in CSCs [103]. TECs may possess stemness properties as they express MDR1 and other stemness markers such as aldehyde dehydrogenase (ALDH), CD90, and stem cell antigen-1 (Sca-1) [104]. These stem cell features of TECs suggest the versatility in their function and continuous availability. 

The survival and function of TECs in the tumor microenvironment can be promoted by the expression of microRNAs (miR-145), which confers on the cells the ability to resist anoikis and become more adhesive by activating ERK1/2 and epigenetic modifications to enhance miR-145 expression [105]. Circulating tumor-associated ECs that express Bcl-2 have been implicated in protecting tumor cells from anoikis and enhancing lung metastasis. For TECs to protect tumor cells in circulation, they should also possess the ability to survive in circulation [106]. Although the study did not address anoikis resistance in TECs, the authors showed that TECs exhibited increased adhesive properties and were Bcl-2-positive, which was similar to the effect of miR145 on anoikis-resistant TECs in our study. Bcl-2 upregulation has been shown to enhance EC survival [107]. These findings may imply that anoikis-resistant TECs could promote tumor metastasis. In fact, we have demonstrated that TECs can elicit metastasis of low-metastatic tumors to the lung by releasing the angiocrine factor biglycan [108]. 

### 5.4. Altered Metabolism

EC metabolism plays a crucial role in the formation of blood vessels. In general, most of the known metabolic–biosynthetic pathways are more activated in TECs than in NECs [109]. Glycolysis regulates the migration and proliferation of tip and stalk ECs, respectively, through the activity of the glycolytic enzyme phosphofructokinase-2/fructose-2,6-bisphosphatase 3 (PFKFB3) [110]. Although normal healthy ECs were shown to have a higher glycolytic level than other healthy cells [110], we and other groups have demonstrated that glycolysis is more activated in TECs [111], conferring on these cells the “hyperglycolytic” label [112,113]. In these hyperglycolytic TECs, PFKFB3 and other glycolytic enzymes are upregulated compared to those in their normal counterparts. Inhibition of PFKFB3 in ECs decreased tumor metastasis by inducing the normalization of the tumor vessels. The vessel structure was improved from a disorganized structure to more matured pericyte-covered, perfused vessels, which supported better drug delivery leading to antimetastatic therapeutic benefits [112]. In addition, cyclooxygenase 2 (COX2) supports the upregulated expression of PFKFB3 and VEGF in TECs to facilitate the higher glycolytic rates in these cells [113]. This role of COX2 in TEC glycolysis may partly explain our previous discovery that COX2 is essential for tumor angiogenesis. We found that COX2 was upregulated in TECs and its inhibition decreased the tumor growth and the recruitment of vascular progenitor cells into tumor vessels. COX2 inhibition further reduced the migration and proliferation of TECs, implying that both the angiogenic activity of resident TECs in the tumor and the recruitment of progenitor cells that can become TECs are impaired by COX2 [114]. The hyperglycolytic nature of TECs may potentially contribute to the overwhelming lactate burden within the tumor and exert various effects on stromal cells. It is interesting to note that somehow TECs survive in these high-lactate environments, and angiogenesis is not impaired. We have reported that in addition to the enzymes that directly influence substrate metabolism, enzymes that maintain intracellular pH at physiological levels significantly influence TEC proliferation. TECs release more lactate extracellularly than NECs, accompanied by an acidification of the culture media. Notably, these TECs displayed an upregulated expression of the pH regulatory enzyme carbonic anhydrase 2 (CAII). CAII in TECs was required for successful proliferation, independent of the metabolic substrate available [111]. Moreover, to maintain their higher proliferative rates, TECs require nucleotide precursors and lipids from serine and lipid biosynthesis pathways, respectively. To support this demand for biomolecules, TECs express higher levels of key pathway enzymes such as D-3-phosphoglycerate dehydrogenase (PHGDH) and phosphoserine aminotransferase 1 (PSAT1) [112] for serine biosynthesis and fatty acid synthase (FASN) [115] for lipid synthesis. Nucleotide biosynthesis is also enhanced in TECs compared to that in NECs [109]. TECs are a robust group of cells that deserve special attention and study in the pursuit of direct, effective tumor angiogenesis therapeutic strategies.

## 6. Utilizing TEC Characteristics to Improve Antiangiogenic Therapy Clinically

### 6.1. Evaluation of Tumor Angiogenesis and Treatment Outcomes

**Figure 3:**
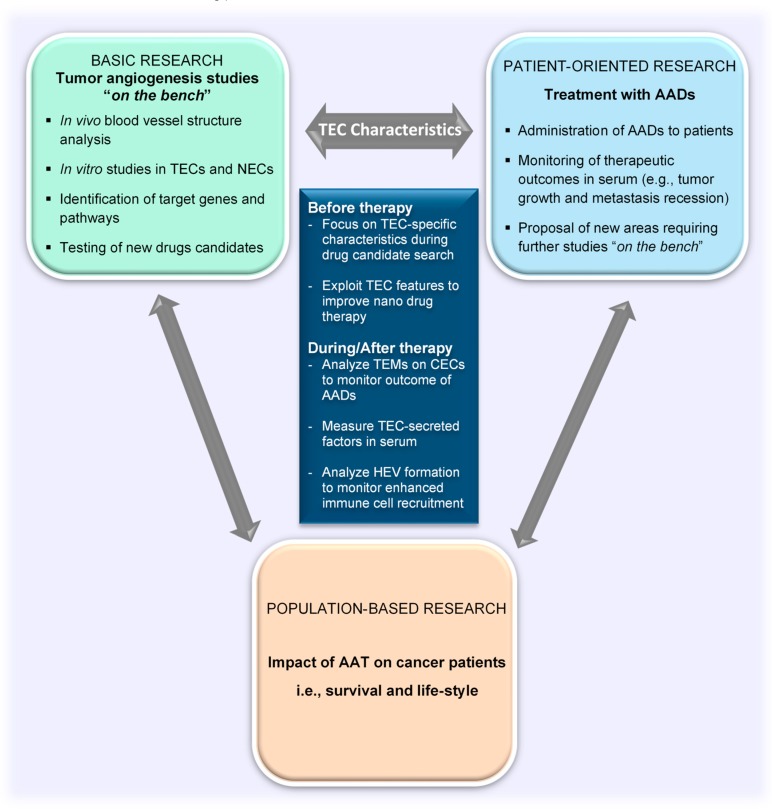
TEC characteristics as a tool to bridge the gap between basic and clinical research in the field of tumor angiogenesis. TEC characteristics as explored in basic research are ideal targets for the development of drugs for clinical applications. Drugs that specifically target TEC characteristics can enhance vessel normalization to allow for better delivery of nanodrugs. Clinical outcomes can be noninvasively evaluated in serum by measuring TEC-secreted factors and characteristics of circulating TECs released from tumors. TEC, tumor endothelial cell; NEC, normal endothelial cell; CEC, circulating endothelial cell; AADs, antiangiogenesis drugs; TEMs, tumor endothelial markers; AATs, antiangiogenic therapies

Having described the characteristics of TECs, the focus of this review will now be turned toward elaborating the contribution of this special groups of cells to improve clinical applications of tumor angiogenesis research findings. We shall address the current methodologies for evaluating the efficacy of antiangiogenesis therapy and some factors that affect the overall therapeutic outcome of angiogenesis inhibition on cancer progression (i.e., tumor angiogenesis, tumor growth, immune cell activity). We shall also suggest the aspects of TEC biology that can bridge the basic and the patient-oriented arms of translational research (Figure 3). 

The endothelium is a major target of therapeutic strategies aimed at eliminating cancers and managing the metastatic spread of tumors. The vasculature in tumors is generally analyzed by the quantification of the microvessel number or area found within the tumor through immunohistochemical analysis after staining common EC markers such as CD31 and CD34. Although these approaches can provide a good idea about the microvessel presence and density within the tumor, they do not adequately inform about the blood vessel formation process or how the vasculature changes in response to therapeutic strategies targeting the tumor cells and/or TECs. For instance, tumor hypoxia is one negative side effect of antiangiogenic therapy that adversely leads to tumor revascularization and aggressive tumor growth [116]; as such, focusing on the alterations in the TECs that are likely to be used to initiate and facilitate posttreatment revascularization, and not only the reduction in microvessel density after therapy, will be more beneficial. To achieve this, it is important to understand the effect of antiangiogenesis drugs on the genes characteristic of proliferating and migrating TECs (e.g., MMP9, VEGFR2, and VEGFA) [96,117]. The reduction or cessation of expression of such TEC-marker genes could be examined, and this will also serve as a way of monitoring drug efficacies. In soft tissue sarcomas, for instance, microvessel density was not a good predictor of disease outcome in patients. VEGF expression in the tissue was found to be a better indicator of local recurrence and metastasis in the patients analyzed in that study [118].

In addition, tumor vessel normalization is a very essential occurrence after antiangiogenesis drug treatment, which holds adequate promise for improving anticancer and cancer immunotherapies. Researchers analyze certain vessel components such as the coverage of perivascular cells (e.g., pericytes [119]) or the expression of certain proteins (e.g., VE-cadherin [120]) to verify the maturity and integrity [121,122,123] of blood vessels. Although these factors can inform about normalization of tumor vessels and a potential improvement in drug delivery [124], they do not adequately describe the functional changes in TECs within the tumor. For instance, there is a “vascular normalization window” (the time point at which the tumor hypoxia is decreased and there is the potential for maximal therapeutic outcome and improved blood vessel structure) [125] which must be realized so that the tumor vessels are not regressed completely by antiangiogenesis drugs or excessive hypoxia occurs with its detrimental outcomes. Jain also proposed the need for an antiangiogenic cocktail for normalization due to the different growth requirements of tumor cells at different stages of progression and the multiplicity of angiogenic pathways and kinases that control the tumor angiogenesis process [27]. Similarly, there is a need for a mixture of genes/molecules that can be used to track the process of vessel normalization or the beginning and entire duration of the vascular normalization window. Current analysis methods demand invasive techniques (e.g., tissue biopsies and surgical resection) to obtain tumor tissues for further investigation [126]. TEC-specific features on ECs (i.e., TEMs, which are found in the plasma or serum of patients with cancer) can be explored to monitor vessel normalization as well as minimize the use of such invasive methods. However, it is necessary to screen a large number of patient samples and check the expression of thousands of genes to come up with such good gene sets. Therefore, TEC-specific genes will serve as a good starting point in this search. 

Despite the considerable ambiguity about the origin of circulating endothelial cells (CECs), they have been proposed as biomarkers for cancer. Mehran et al. described the importance of TEMs expressed on CECs; they suggested that circulating TEM (+) ECs would be more specific for cancers and therefore better for evaluating the response to therapy, because TEMs can identify the tumor-derived CECs better (Figure 3). The authors were able to show a reduction in the circulating TEM (+) ECs after both antiangiogenic therapy and surgical resection of tumors [127]. However, their study described primarily TEM7 and CD276 as markers for circulating TECs, which may not be sufficient. Further studies are required to identify and create TEC gene sets to be analyzed for proper monitoring of cancer progression and therapeutic outcomes in patients with cancer.

Companion diagnostics are still emerging, and biomarkers are required to develop this area of cancer therapy. In addition to TEMs expressed on CECs, the measurement of molecules secreted into blood will be beneficial. Proteoglycans such as biglycan and endocan could be monitored in the blood of patients with cancer as potential biomarkers for companion diagnostics before, during, and after antiangiogenic therapy [128]. Both biglycan and endocan expressions are upregulated in activated ECs, including TECs in tumors. We have reported that TECs, particularly those isolated from highly metastatic melanoma, possess significantly upregulated biglycan mRNA and protein levels [108,129]. In addition, we found the levels of plasma biglycan to be elevated in patients with cancer compared to those in healthy controls [108]. Endocan expression was observed in ECs in glomeruloid vessels and proliferating microvessels in high-grade glioblastoma tumors [130]. Similar to biglycan, serum levels of endocan have been reported to be also higher in patients with cancers of the lung [131], kidney [132], and liver [133] than in controls.

Certain imaging techniques, including computed tomography (CT), magnetic resonance imaging (MRI), emission computed tomography (ECT), and dynamic contrast-enhanced ultrasonography (DCE-US), have also been used to monitor the tumor vasculature. Although they have yielded some good results, they are limited by various factors, including scanning solutions, contrast medium, image analysis software and personal bias, type of tumor, and the absence of appropriate controls and standards [134]. Additional research and a unified standard must be developed to enhance the sensitivity and reliability of these techniques because they hold much promise to a noninvasive evaluation of tumor vascular normalization and monitoring of changes in vessels during therapy.

### 6.2. Enhancing the Therapeutic Benefits of Nanoparticles and Immune Checkpoint Inhibitors

TECs produce other soluble factors and chemokines that affect the tumor cells and other stromal cells, as well as the TECs themselves. Some of these factors include CXCL12, IL3, IL6, IL8, lysyl oxidase, granulocyte colony-stimulating factor, transforming growth factor β, among others [97]. TECs express receptors such as CXCR7, endoglin, integrins (e.g., αv integrins), and VEGFR2. Integrins, endoglin, and VEGFR2 have been used in antiangiogenic nanodrug therapies and are still in development [135]. Using these TEC-specific nanodrugs indicates a shift into a regime of antiangiogenic drugs with less off-target effects.

The enhanced permeability and retention (EPR) effect is a feature of tumors that allows the delivery and retention of macromolecular drugs due to the leakiness of blood vessels and the lack of adequate drainage through the lymphatics [136]. The contribution of the abnormal tumor vasculature to the EPR effect and its role in delivery of nanoparticles have been well documented. Reduced pericyte coverage that renders the interendothelial barriers accessible is one such feature of the abnormal vasculature that enhances the EPR effect. The tumor endothelium facilitates the detachment of pericytes by expressing angiopoietin 2 (Ang2); it has been reported that Ang2 antagonizes the binding of Ang1 to the Tie2 receptors on TECs [137]. TEC heterogeneity [97] is an unexplored feature of TECs that may contribute significantly to the EPR effect in tumors. TECs originating from highly metastatic tumors express high levels of VEGF and VEGFR2 [96], and these features can be advantageous to the EPR effect because they promote chaotic blood vessel formation. In addition, the hyperglycolytic metabolism of TECs has been shown to decrease the expression of VE-cadherin in vessels, leading to the formation of abnormal blood vessels. Inhibition of PFKFB3 resulted in more VE-cadherin deposition and vessel normalization with better therapeutic outcomes [112]. Although these TEC characteristics have been proposed as therapeutic targets, with the development of drugs (e.g., Tie2/Ang2), a balance needs to be found during therapy to maintain a better window that will both improve the tumor vessel structure and still support drug delivery through the use of nanoparticles against cancer cells.

In addition to serving as the channel through which drugs reach the tumor, tumor blood vessels also become either the gateway for immune cell infiltration or an antagonist for immune cell activity within the tumor. TECs upregulate the production of prostaglandin E2 (PGE2) upon induction by tumor-derived VEGF, and this mechanism can suppress CD4+ and CD8+ T-cell secretion of IL-2 and IFγ, thereby inhibiting immune functions [138]. Both angiogenesis and immune cells have been the focus of cancer therapy in recent times. Improving cancer immunotherapy will require a rise in the infiltration of immune cells. High endothelial venules (HEVs) are structurally distinct endothelia of the postcapillary venules. HEVs are found in lymphoid tissues (e.g., lymph nodes and Peyer’s patches) and are characterized by the presence of tall and cuboidal ECs surrounded by a thick basal lamina and prominent perivascular sheath. They allow the extravasation of lymphocytes from the blood [139,140]. An increase in the number of HEVs in tissues, including tumors, is likely to enhance the infiltration of lymphocytes. HEV precursor ECs may include immature ECs and endothelial precursor cells. In the past decade, we have shown that TECs are made up of a heterogenous population of cells [96,97,104,141], and a recent study conducted by Goveia et al. used single-cell RNA sequencing and orthogonal multiomics approaches to demonstrate the heterogeneity of TECs at the single-cell level using murine and human samples [142]. The authors reported that TECs are made up of a high quantity of immature ECs. The presence of immature ECs and EPCs in TEC populations suggests that TECs have the potential to transform into HEVs within the tumor to facilitate immune cell infiltration and cancer treatment (Figure 3). In fact, a study demonstrated an increase in HEV formation from the tumor vasculature after treatment with anti-VEGFR2 and anti-PD1 antibodies in polyoma middle T oncoprotein breast cancer and Rip1-Tag2 pancreatic neuroendocrine tumor models. The authors of that study observed morphological changes such as thickened vessels with plump ECs in the tumor vasculature and a positive expression of MECA79 (an established HEV antibody) [143]. 

## 7. Conclusions

Translational research has come to stay and all efforts to promote this field of cancer research are important. The focus of translational research for cancer targeting through tumor vessels would have to be shifted from the generalized canopy of tumor angiogenesis to a narrower focus on TEC biology. Studies on TEC biology and TEC-specific characteristics (i.e., on the “bench”) will facilitate the journey toward finding the ideal angiogenic agent that would yield less off-target effects in patients (i.e., at the “bedside”).

## Abbreviations

AADs	Antiangiogenesis drugs
AATs	Antiangiogenic therapy
ABC	ATP-binding cassette transporter family
ACRT	Association for Clinical Research Training
Ang 2	Angiopoietin 2
bFGF	basic fibroblast growth factor
CAII	Carbonic anhydrase 2
CECs	Circulating endothelial cells
COX2	Cyclooxygenase 2
CSC	Cancer stem cells
CT	Computed tomography
DCE-US	Dynamic contrast-enhanced ultrasonography
EC	Endothelial cells
ECT	Emission computed tomography
EGFR	Epidermal growth factor receptor
EPC	Endothelial progenitor cells
EPR	Enhanced permeability and retention
FASN	Fatty acid synthase
HEVs	High endothelial venules
IFN	Interferon
IGF-1	Insulin-like growth factor
IL	Interleukin
mTOR	Mammalian target of rapamycin
miR	MicroRNA
MDR1	Multidrug resistance protein 1
MMPs	Matrix metalloproteinases
MRI	Magnetic resonance imaging
NEC	Normal endothelial cells
PDGF	Platelet-derived growth factor
PECAM1	Platelet endothelial cell adhesion molecule 1
PFKFB3	Phosphofructokinase-2/fructose-2,6-bisphosphatase 3
PGE2	Prostaglandin E2
PHGDH	D-3-phosphoglycerate dehydrogenase
PSAT1	Phosphoserine aminotransferase 1
Sca-1	Stem cell antigen-1
SDF-1	Stromal cell-derived factor-1
TAMs	Tumor-associated macrophages
TEC	Tumor endothelial cells
TEMs	Tumor endothelial markers
TKIs	Tyrosine kinase inhibitors
TP53	Tumor protein p53
VEGF	Vascular endothelial growth factor
VEGFR	Vascular endothelial growth factor receptor
